# Target‐site and non‐target‐site mechanisms confer multiple herbicide resistance in waterhemp ( *Amaranthus tuberculatus* ) accessions from Wisconsin

**DOI:** 10.1002/ps.70672

**Published:** 2026-03-02

**Authors:** Felipe A. Faleco, Isabel S. Werle, Alexander J. Lopez, Damilola A. Raiyemo, Patrick J. Tranel, Jed B. Colquhoun, Mark J. Renz, David E. Stoltenberg, Rodrigo Werle

**Affiliations:** ^1^ Department of Plant and Agroecosystem Sciences University of Wisconsin‐Madison Madison WI USA; ^2^ Department of Crop Sciences University of Illinois Urbana‐Champaign IL USA

**Keywords:** 2,4‐D, GST, mesotrione, metabolic resistance, P450

## Abstract

**BACKGROUND:**

A preliminary screening identified a multiple herbicide‐resistant waterhemp, *Amaranthus tuberculatus* (Moq.) Sauer, accession (A101) exhibiting resistance to 2,4‐D and atrazine despite no prior exposure to these herbicides. Therefore, our objective was to characterize resistance to 2,4‐D, atrazine, glyphosate, fomesafen, and mesotrione in A101, along with two additional multiple herbicide‐resistant accessions (A75 and A103).

**RESULTS:**

A101 exhibited low to medium levels of resistance to all five herbicides evaluated (ranging from 1.8‐fold for mesotrione to 8.5‐fold for fomesafen). Both A75 and A103 also had multiple resistance to glyphosate and atrazine, with A75 and A103 additionally resistant to 2,4‐D and fomesafen, respectively. Amplification of *EPSPS* and the P106S substitution accounted for some of the glyphosate resistance, and some of the fomesafen resistance was explained by the G210 deletion in the target enzyme. Moreover, the use of cytochrome P450 monooxygenases (P450s) and glutathione *S*‐transferases (GSTs) inhibitors indicated that non‐target‐site resistance (NTSR) mechanisms also contribute to at least some of the resistance traits.

**CONCLUSION:**

Metabolic resistance to 2,4‐D and atrazine suggests that the use of other herbicides may have contributed to the selection of enhanced P450s and GSTs activity in A101 accession. To our knowledge, this is the first report of P450s associated with atrazine resistance in *A. tuberculatus* globally. A101 is the first confirmed case of *A. tuberculatus* resistance to hydroxyphenyl pyruvate dioxygenase inhibitors in Wisconsin, exhibiting a low‐level resistance likely associated with P450s and GSTs activity. Our results suggest the coexistence of target‐site resistance and NTSR mechanisms associated with glyphosate resistance in A101. © 2026 The Author(s). *Pest Management Science* published by John Wiley & Sons Ltd on behalf of Society of Chemical Industry.

## Graphical abstract

The A101 accession is the first confirmed case of hydroxyphenyl pyruvate dioxygenase resistance in *Amaranthus tuberculatus* in Wisconsin, and the first report of P450s associated with atrazine resistance in *A. tuberculatus* globally.

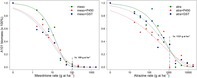

## 1 INTRODUCTION

Waterhemp, *Amaranthus tuberculatus* (Moq.) Sauer, is one of the most common and troublesome weed species in the Midwest USA, particularly in corn (*Zea mays* L.) and soybean (*Glycine max* (L.) Merr.).^1^, ^2^ Moreover, the evolution of herbicide resistance presents a significant challenge to *A. tuberculatus* management. In the USA, *A. tuberculatus* has evolved resistance to herbicides spanning seven sites of action (SOAs): acetolactate synthase (ALS), auxin mimics, photosynthesis at photosystem II–serine 264 binders (PSII), enolpyruvyl shikimate phosphate synthase (EPSPS), protoporphyrinogen oxidase (PPO), very long‐chain fatty acid synthesis (VLCFA), and hydroxyphenyl pyruvate dioxygenase (HPPD).^3^, ^4^ In Wisconsin, *A. tuberculatus* resistance to ALS and EPSPS inhibitors is widespread, whereas resistance to auxin mimics, and to PSII and PPO inhibitors is present to a lesser extent.^5^, ^6^, ^7^

Herbicide resistance in weedy species is classified as either target‐site resistance (TSR) or non‐target‐site resistance (NTSR). TSR impairs herbicide binding at the target site through multiple mechanisms, including increased gene expression and amino acid substitutions or deletions.^4^, ^8^ Conversely, NTSR typically reduces the concentration of herbicide reaching the target site and may occur through multiple mechanisms, including decreased herbicide absorption and translocation, as well as enhanced herbicide detoxification by enzymes, such as cytochrome P450 monooxygenases (P450s) and glutathione *S*‐transferases (GSTs).^4^, ^8^, ^9^

P450s and GSTs are important groups of enzymes that catalyze the metabolism of several organic compounds, including endogenous and xenobiotic compounds in living organisms.^9^, ^10^, ^11^, ^12^, ^13^ These enzymes have shown the ability to metabolize a wide range of herbicides, often leading to scenarios of cross‐resistance (a single mechanism that confers resistance to different herbicides) or multiple resistance (distinct mechanisms conferring resistance to multiple herbicides) in weeds.^9^, ^14^, ^15^ For example, a P450 from the CYP81A subfamily is known to metabolize 18 herbicides from 13 chemical families in late watergrass (*Echinochloa phyllopogon* (Stapf) Koso‐Pol), including acetyl CoA carboxylase (ACCase), ALS, PSII, phytoene desaturase, deoxy‐d‐xylulose phosphate synthase (DOXPS), PPO, and HPPD inhibitors.^16^ In *A. tuberculatus*, P450s have been associated with resistance to auxin mimics,^17^, ^18^, ^19^, ^20^ and to PPO,^21^ VLCFA,^22^ and HPPD inhibitors.^23^, ^24^, ^25^ Meanwhile, GSTs have been reported to be associated with auxin mimics,^26^ and with PSII,^25^, ^27^, ^28^ VLCFA,^22^ and HPPD inhibitor resistance.^29^

In the summer of 2019, a suspected multiple herbicide‐resistant *A. tuberculatus* population from Dane County, Wisconsin, USA (A101 accession) was reported to the Wisconsin Cropping Systems Weed Science Program. A preliminary glasshouse screening identified A101 exhibiting reduced sensitivity to multiple herbicides, including 2,4‐D, atrazine, glyphosate, fomesafen, and mesotrione (data not shown). Interestingly, this field was in a conventional seed corn and soybean rotation for more than 10 years and had no historical use of atrazine, particularly because it is located in an atrazine prohibition area, according to the State of Wisconsin.^30^ Moreover, to our knowledge, 2,4‐D use in this field has been exclusively for winter annual weed control in the fall and has not been directly applied to *A. tuberculatus* plants. Therefore, our objective was to characterize resistance to these herbicides in the A101 accession, along with two additional multiple herbicide‐resistant accessions (A75 and A103).^6^ We hypothesized that resistance in these accessions was driven by both TSR and NTSR mechanisms.

## 2 MATERIALS AND METHODS

### 2.1 *Amaranthus tuberculatus* seed collection and research sites

Seed samples from *A. tuberculatus* plants were harvested in the fall of 2020 and pooled among plants in the same field to compose the A101 accession (Dane County, WI, USA). Two additional multiple herbicide‐resistant accessions were included in the experiments—A75, harvested in 2018 from Fond du Lac County, WI, USA^6^ and A103, harvested in 2021 from the same geographic location as A20 (Brown County, WI, USA).^6^ A susceptible accession (A82), harvested in 2018 from Chippewa County, WI, USA,^6^ was included for treatment comparisons in the metabolic herbicide resistance experiments. Because of limited seed availability of the A82 accession, an F_1_ generation of A82 parent plants was grown under glasshouse conditions, as described below, to compose the A106 accession. The A106 accession was used as the susceptible accession in the ametryne and TSR experiments. An additional susceptible accession (A92), harvested in 2021 from Dodge County, WI, USA^5^ was included as the control group in the differential gene expression (DGE) analysis in the TSR experiments.

Seeds from each *A. tuberculatus* accession were threshed from other plant material and cleaned using a seed blower separator (Oregon Seed Blower, Hoffman Manufacturing, Inc., Jefferson, OR, USA). To increase germination rate, seeds from each accession were placed in a glass container, floated on a thin layer of water, and stratified in the dark at 5 °C for 2 weeks. After this period, seeds were washed with water using a soil sieve mesh to retain the seeds and dried on paper towels at room temperature for 24 h.^6^ Seeds were placed in plastic bags and stored at 5 °C until the onset of experiments.

The metabolic herbicide resistance experiments (2022–2024) and the ametryne experiment (2025) were conducted at the University of Wisconsin‐Madison Walnut Street Greenhouses, Madison, WI, USA. The TSR experiments (2024–2025) were conducted at the University of Illinois‐Urbana Champaign, IL, USA.

### 2.2 Assessment of metabolic herbicide resistance in *A. tuberculatus* accessions

Dose–response glasshouse experiments were conducted to determine the presence of 2,4‐D, atrazine, glyphosate, fomesafen, and mesotrione metabolic resistance in A75, A101, A103. The A82 (susceptible accession) was included for treatment comparisons. The experiments were conducted in a completely randomized design (CRD), with four replications per treatment, and were repeated over time (two experimental runs). Each herbicide was evaluated in separate experiments under three conditions: without pre‐treatment (herbicide alone), herbicide applied after a pre‐treatment with a P450 inhibitor (malathion; herbicide + P450), and herbicide applied after a pre‐treatment with a GST inhibitor [4‐chloro‐7‐nitrobenzofurazan (NBD‐Cl); herbicide + GST]. Herbicide rates were 0× [herbicide non‐treated control (HNTC)], 0.015×, 0.031×, 0.062×, 0.125×, 0.25×, 0.5×, 1×, 2×, 4×, 8×, and 16× the label rate (Table 1). The P450‐ and GST‐inhibitor rates, as well as the adjuvant rates, were maintained at 1× (Table 1).

**Table 1:** Herbicides, P450‐ and glutathione *S*‐transferase inhibitor evaluated in the assessment of metabolic herbicide resistance in *Amaranthus tuberculatus* accessions experiment

				Rate^§^			
Active ingredient	Trade name	Formulation^†^	WSSA SOA^‡^	Active ingredient	HSOC^†^	AMS^†^	Herbicide manufacturer
				(g ai or ae ha^−1^)	(v/v %)	(g ha^−1^)	
2,4‐D	Enlist One®	3.8 SL	AM (4)	1065	—^¶^	1429	Corteva Agriscience, LLC, Indianapolis, IN, USA
Atrazine	Aatrex® 4 L	4 SL	PSII (5)	1121	0.8	—	Syngenta Crop Protection, LLC, Greensboro, NC, USA
Glyphosate	Roundup Powermax® 3	4.8 SL	EPSPS (9)	841	—	1429	Bayer CropScience, LP, St. Louis, MO, USA
Fomesafen	Flexstar®	1.88 SL	PPO (14)	263	0.5	1429	Syngenta Crop Protection, LLC, Greensboro, NC, USA
Mesotrione	Callisto®	4 SC	HPPD (27)	105	0.5	1429	Syngenta Crop Protection, LLC, Greensboro, NC, USA
Malathion	Fyfanon® 57% EC	5 EC	N/A	2000	—	—	FMC Corporation, Philadelphia, PA, USA
NBD‐Cl^†^	N/A	98%	N/A	270	—	—	Sigma‐Aldrich, Inc., St. Louis, MO, USA

^†^ NBD‐Cl, 4‐chloro‐7‐nitrobenzofurazan; SL, soluble liquid; SC, soluble concentrate; EC, emulsifiable concentrate; HSOC, high surfactant oil concentrate; AMS, ammonium sulfate.

^‡^ Weed Science Society of America (WSSA) Herbicide Site of Action (SOA): AM, auxin mimics (Group 4); PSII, photosynthesis at PSII–serine 264 binders (Group 5); EPSPS, enolpyruvyl shikimate phosphate synthase (Group 9); PPO, protoporphyrinogen oxidase (Group 14); HPPD, hydroxyphenyl pyruvate dioxygenase (Group 27).

^§^ The herbicide and adjuvant rates were determined based on the respective label crop use directions for post‐emergence application in corn or soybean, and recommendations for controlling *A. tuberculatus* when specified. Malathion and NBD‐Cl rates were determined based on previously published research.

^¶^ —, adjuvant was not included; N/A, Not Applicable.

*Amaranthus tuberculatus* seeds were planted 1.5 cm deep in potting mix (Promix® HP Mycorrhizae, Premier Tech Horticulture, QC, Canada) contained in 8600‐mL plastic flats (1020 Standard Full Depth Vacuum Flat; The HC Companies, Twinsburg, OH, USA). Seedlings at the four true‐leaf stage were transplanted into 656‐mL pots (D40H Deepots™; Stuewe & Sons Inc, Tangent, OR, USA) filled with potting mix. The experimental unit was one seedling per pot. The P450 and GST inhibitors were applied prior to herbicide applications using a single‐nozzle research track spray chamber (DeVries Manufacturing, Hollandale, MN, USA) equipped with a AI9502EVS nozzle (extremely coarse droplet size at 276 kPa; TeeJet Technologies, Spraying Systems Co., Wheaton, IL, USA). For 2,4‐D and mesotrione, both P450 and GST inhibitors were applied 48 h before herbicide. For atrazine, glyphosate, and fomesafen, the methodology was slightly adjusted to be similar to that reported in current literature, with P450 and GST inhibitors applied 1 and 48 h before the herbicide, respectively. This method was adapted from previously published work.^20^, ^25^, ^31^, ^32^

Herbicides were applied to plants 5–10 cm in height using the spray chamber described above equipped with a AI9502EVS nozzle for 2,4‐D and a DG9502EVS nozzle (medium droplet size at 276 kPa; TeeJet Technologies) for other herbicides. A carrier volume of 140 L ha^−1^ and pressure of 276 kPa were used for all applications. Plants were maintained in the glasshouse at 20–30 °C (min–max), with a forced ventilation system. Relative humidity ranged from 20% to 100% and was not controlled, allowing for natural variation in the glasshouse. Natural lighting was supplemented with artificial lighting from 1000 W high‐pressure sodium light bulbs (130 000 lm; LU1000; Ledvance LLC, Wilmington, MA, USA), providing a 16:8 h light/dark photoperiod. Plants were top‐watered daily and fertigated weekly with 20–10–20 water‐soluble fertilizer (Peters Professional, ICL Fertilizers, Dublin, OH, USA) delivering 500 ppm of nitrogen (N) and potassium (K), respectively, and 250 ppm of phosphorus (P).

At 21 days after treatment (DAT), aboveground biomass was harvested and forced‐air dried at 50 °C to constant mass. Biomass data were converted to percent biomass relative to the respective HNTC (herbicide alone, herbicide + P450, or herbicide + GST) using Eqn (1), adapted from a previous study.^33^

(1) $$\text{Biomass}\;\left(\%\right)\;=\;\frac{\mathrm{BEU}}{\bar{\text{BHNTC}}}\;\times \;100$$

where BEU is the biomass of the experimental unit and $\bar{\text{BHNTC}}$ is the biomass mean of the respective HNTC.

Dose–response models were fitted to percent biomass data using the *drm* function in the *drc* package v.3.0‐1^34^ in *R* v.4.4.1^35^ and RStudio v.2024.9.0.375.^36^ The model for each analysis was selected based on the lowest Akaike information criterion from the *mselect* function (*drc* package). The two‐parameter Weibull type 1 model (W1.2; Eqn (2)) was used for all herbicides, except for glyphosate, for which the three‐parameter Weibull type 2 model (W2.3; Eqn (3)) was used.

(2) $$f\left(x\right)\;=\;\exp\left(-\exp\left(b\left(\log\left(x\right)-\log\left(e\right)\right)\right)\right)$$

where b is the relative slope around the inflection point e.^34^, ^37^

(3) $$f\left(x\right)\;=\;0\;+\;\left(d-0\right)\left(1-\exp\left(-\exp\left(b\left(\log\left(x\right)-\log\left(e\right)\right)\right)\right)\right)$$

where d is the upper limit, and b is the relative slope around the inflection point e.^34^, ^37^

To identify herbicide‐resistant accessions, dose–response models were fitted comparing accessions based on the herbicide‐alone treatments. In asymmetrical models, such as W1.2 and W2.3, the parameter e (inflection point) does not correspond to the ED_50_ (here, defined as the estimated herbicide rate required to reduce percent biomass by 50% relative to the respective HNTC). Therefore, the absolute ED_50_ (rather than the commonly used relative ED_50_)^37^ was estimated using the *ED.drc* function (*drc* package). The *EDcomp* function (*drc* package), which performs a Student's *t*‐test, was used to estimate the resistance index (RI) by comparing the absolute ED_50_ of each accession *versus* the absolute ED_50_ of the susceptible accession (A82).

To assess the presence of metabolic resistance in each resistant accession, dose–response models were fitted comparing herbicide alone, herbicide + P450, and herbicide + GST treatments for each accession. As described above, the *EDcomp* function was used to estimate the RI by comparing the absolute ED_50_ of the herbicide + P450 or herbicide + GST *versus* the absolute ED_50_ of the respective herbicide‐alone treatment. Because our objective was to assess the response of the *A. tuberculatus* accessions evaluated herein without generating plants that were homozygous for resistance, a significance level of *α* = 0.1 was used in all comparisons.

### 2.3 Assessment of ametryne resistance in *A. tuberculatus* accessions

Ametryne is structurally similar to atrazine and has the same SOA (PSII inhibitor), but because it has a thiomethyl group instead of a chlorine attached to the heterocyclic ring,^38^ it can undergo different detoxification pathways.^39^ Specifically, because a GST can catalyze the replacement of chlorine with glutathione, a GST may conjugate glutathione to atrazine, but not to ametryne.^40^, ^41^ Therefore, an *A. tuberculatus* plant resistant to atrazine but susceptible to ametryne suggests the association of GSTs in atrazine detoxification.^39^

Following the assessment of metabolic herbicide resistance experiments, which identified P450s but not GSTs associated with atrazine resistance in accessions A75, A101, and A103 (Table 2), a dose–response glasshouse experiment was conducted to quantify the sensitivity of these accessions to ametryne. The A106 accession was included for treatment comparisons (susceptible accession). The experiment was organized in a CRD with four replications per treatment and repeated over time (two experimental runs). Herbicide rates were 0×, 0.015×, 0.031x, 0.062×, 0.125×, 0.25×, 0.5×, 1×, 2×, 4×, 8×, and 16× the label rate of ametryne [Evik® DF; Syngenta Crop Protection, LLC, Greensboro, NC, USA; 1× = 1769 g ai ha^−1^ + 0.5 v/v % high surfactant oil concentrate (HSOC)]. *Amaranthus tuberculatus* seeds were planted and seedlings transplanted as described above. The experimental unit was one seedling per pot. Herbicides were applied when plants reached 5–10 cm in height using the spray chamber, carrier volume, and pressure described above, equipped with a DG9502EVS nozzle (TeeJet Technologies). Plants were maintained in the glasshouse with the same conditions as described above. At 21 DAT, aboveground biomass was harvested, forced‐air dried, and biomass data were converted to percent using Eqn (2) as described above.

**Table 2:** Dose–response models output comparing herbicide alone, herbicide + P450, and herbicide + glutathione *S*‐transferase (GST) for A75, A101, and A103 (multiple herbicide resistant) and A82 (susceptible) *Amaranthus tuberculatus* accessions from Wisconsin at 21 days after treatment

Accession	Treatment	ED_50_ percent biomass (g ai or ae ha^−1^)^†^	ED_50_ RI^‡^	ED_50_ RI *P* value
A75	2,4‐D + P450	107.3 (±17.8)	—^§^	0.3943
	2,4‐D + GST	93.3 (±19.5)	—	0.1487
	2,4‐D	126.7 (±16.7)	—	—
	Atrazine + P450	513.0 (±119.1)	0.60	0.0535
	Atrazine + GST	662.9 (±151.2)	—	0.3906
	Atrazine	860.3 (±224.4)	—	—
	Glyphosate + P450	2847.8 (±722.5)	—	0.7069
	Glyphosate + GST	3789.6 (±1194.7)	—	0.7102
	Glyphosate	3226.3 (±792.2)	—	—
A101	2,4‐D + P450	177.7 (±22.4)	0.59	< 0.0001
	2,4‐D + GST	124.8 (±19.0)	0.42	< 0.0001
	2,4‐D	299.9 (±28.0)	—	—
	Atrazine + P450	134.8 (±40.0)	0.32	< 0.0001
	Atrazine + GST	298.2 (±63.7)	—	0.1252
	Atrazine	426.4 (±76.8)	—	—
	Glyphosate + P450	3202.9 (±796.1)	—	0.3289
	Glyphosate + GST	856.7 (±274.3)	0.39	< 0.0001
	Glyphosate	2192.0 (±451.1)	—	—
	Fomesafen + P450	41.0 (±9.0)	—	0.6222
	Fomesafen + GST	41.2 (±8.7)	—	0.6267
	Fomesafen	47.0 (±9.3)	—	—
	Mesotrione + P450	6.0 (±0.8)	0.53	< 0.0001
	Mesotrione + GST	4.9 (±0.8)	0.43	< 0.0001
	Mesotrione	11.3 (±13.3)	—	—
A103	Atrazine + P450	704.8 (±144.3)	0.37	< 0.0001
	Atrazine + GST	1981.7 (±345.5)	—	0.8585
	Atrazine	1888.3 (±374.4)	—	—
	Glyphosate + P450	628.6 (±131.9)	—	0.3568
	Glyphosate + GST	687.9 (±196.0)	—	0.6223
	Glyphosate	815.2 (±198.3)	—	—
	Fomesafen + P450	27.1 (±3.5)	2.08	0.0234
	Fomesafen + GST	32.1 (±5.1)	2.47	0.0155
	Fomesafen	13.0 (±2.4)	—	—
A82	Atrazine + P450	104.9 (±18.1)	0.69	0.0522
	Atrazine + GST	154.8 (±28.6)	—	0.9552
	Atrazine	152.7 (±24.0)	—	—
	Mesotrione + P450	4.3 (±0.6)	0.68	0.0156
	Mesotrione + GST	5.6 (±0.7)	—	0.5112
	Mesotrione	6.3 (±0.8)	—	—

^†^ ED_50_ percent biomass, estimated herbicide rate that decreased percent biomass by 50% compared with the respective herbicide non‐treated control.

^‡^ ED_50_ RI, for each accession, the resistance index (RI) was calculated by dividing the ED_50_ of the herbicide + P450 or herbicide + glutathione *S*‐transferase by the ED_50_ of the respective herbicide alone.

^§^ —, the ED_50_ RI and ED_50_ RI *P* value were not calculated for the herbicide alone or when ED_50_ RI *P* value was not significant by Student's *t*‐test (*α* = 0.1).

Standard errors are shown in parentheses.

To identify ametryne‐resistant accessions, a W1.2 (Eqn (2)) dose–response model was fitted to percent biomass following the same procedures described above. The *EDcomp* was used to estimate the RI by comparing the absolute ED_50_ of each accession *versus* the absolute ED_50_ of the susceptible accession (A106), as described above (*α* = 0.1).

### 2.4 Assessment of target‐site herbicide resistance in *A. tuberculatus* accessions

To determine the presence of known TSR mutations conferring resistance to atrazine (PSII D1 S264G substitution),^42^ glyphosate (EPSPS P106S substitution),^43^ and fomesafen (PPO2 ΔG210 deletion and R128G substitution)^44^, ^45^ in A75, A101, and A103 accessions, the sequence and expression of targets were analyzed using an RNA sequencing approach. Furthermore, several additional well‐characterized TSR mutations reported in other species in the herbicide targets were surveyed (Supporting Information, Table S1). Currently, no known mutations have been reported to confer TSR to 2,4‐D in *A. tuberculatus* and the suite of genes encoding proteins with auxin‐binding properties have not yet been identified in this species, thus TSR mutations to this herbicide were not surveyed. A106 (susceptible accession) was included for treatment comparisons. Leaf tissues were sampled from 15 untreated *A. tuberculatus* seedlings of each accession, immediately frozen in liquid nitrogen, and stored at −80 °C until extraction. Total RNA was then extracted using a TRIzol‐based method^46^ followed by treatment with DNase I (Invitrogen) to eliminate contaminating DNA.

After extraction, three RNA pools were created for each accession, each composed of equal amounts of RNA from 5 plants (15 plants total per accession). The RNA quality and quantity of the pooled samples were assessed using gel electrophoresis (1% agarose) and a NanoDrop 1000 spectrophotometer (Thermo Fisher Scientific, Madison, WI, USA), respectively. The RNA samples were then sent to the Roy J. Carver Biotechnology Center at the University of Illinois for Illumina library construction and sequencing. Libraries were prepared with the WatchMaker mRNAseq Prep Poly A+ selection kit (Watchmaker Genomics, Boulder, CO, USA) and sequenced on an Illumina NovaSeq X 25B lane generating 150‐bp paired‐end reads. Sequenced read quality was assessed using FastQC v.0.12.0^47^ and summarized with MultiQC v.1.12.^48^

The occurrence of TSR to PSII, EPSPS, and PPO inhibitors in the pooled samples was determined by aligning the raw reads to a recently available reference genome for *A. tuberculatus* ^49^ using STAR v.2.710b.^50^ Mutation sites reported to confer herbicide resistance in the *psbA*, *EPSPS*, *PPX1*, and *PPX2* genes associated with herbicide resistance in multiple weed species were first identified through a local BLASTn search using available resistant alleles as the query (Supporting Information, Table S1). Read alignments at these loci were then visually inspected and allelic frequencies were calculated to identify the presence and frequency of TSR mutations using the Integrative Genomics Viewer v.2.11.9 genome browser.^51^ To discriminate low‐frequency variants from sequencing error and noise, a minor allele frequency threshold of 0.05 was used.

DGE analysis was also conducted to determine whether increased expression of any herbicide target genes were associated with resistance, as commonly reported with glyphosate resistance.^4^, ^8^ Resistance alleles in the *PPX2* (ΔG210) and *EPSPS* (P106S) genes were detected at low frequency in pools P2 and P3 of A106 (susceptible accession), suggesting that this accession is segregating for both PPO and glyphosate resistance (Table 3). Therefore, only P1 from accession A106 was used as a susceptible control for reference in calling differentially expressed genes in the DGE analysis. To resolve this, three additional RNA pools from an additional *A. tuberculatus* glyphosate‐susceptible control accession (A92)^5^ were created following the same procedures as above and grouped with the A106 P1 sample to represent the susceptible control group for DGE analysis. Reads were counted using the Subread package featureCounts^52^ and genes with ≥1 count per million were normalized using the trimmed mean of M‐values method. Read counts were fit to a negative binomial quasi‐likelihood model in edgeR^53^ and genes with a fold‐change (FC) > 1.2 and false discovery rate (FDR) < 0.05 were considered differentially expressed.

**Table 3:** Frequency of resistance alleles in *Amaranthus tuberculatus* accessions at mutation sites reported to confer resistance to photosystem II (PSII), enolpyruvyl shikimate phosphate synthase (EPSPS), and protoporphyrinogen oxidase (PPO) inhibitors

Accession	Pool ID^†^	PSII	EPSPS	PPO	
		S264G	P106S	ΔG210	R128G
A75	P1	0.000	0.000	0.000	0.000
	P2	0.000	0.000	0.000	0.000
	P3	0.000	0.000	0.000	0.000
A101	P1	0.000	0.000	0.346	0.000
	P2	0.000	0.000	0.291	0.000
	P3	0.000	0.174	0.364	0.000
A103	P1	0.000	0.206	0.124	0.000
	P2	0.000	0.305	0.275	0.000
	P3	0.000	0.140	0.271	0.000
A106	P1	0.000	0.000	0.000	0.000
	P2	0.000	0.255	0.423	0.000
	P3	0.000	0.224	0.229	0.000

^†^ Three separate RNA pools were created for each accession by mixing equal amounts of RNA sampled from five *Amaranthus tuberculatus* seedlings.

## 3 RESULTS

### 3.1 Assessment of metabolic herbicide resistance in *A. tuberculatus* accessions

The A75 accession exhibited an ED_50_ of 126.7 g acid equivalent (ae), Eaton, CO, United States,  ha^−1^ for 2,4‐D (RI = 1.3, *P* = 0.0712) (Table 4), an ED_50_ of 860.2 g active ingredient (ai) ha^−^^1^ for atrazine (RI = 5.6, *P* = 0.0074), and an ED_50_ of 3221.0 g ae ha^−1^ for glyphosate (RI = 10.3, *P* = 0.0017). For A75, P450s inhibition reduced atrazine ED_50_ by 40% (513.0 g ai ha^−1^, *P* = 0.0535) (Table 2) compared with atrazine alone. However, the inhibition of P450s or GSTs did not change 2,4‐D and glyphosate ED_50_ for A75.

**Table 4:** Dose–response model output comparing the A75, A101, and A103 (multiple herbicide resistant) and A82 (susceptible) *Amaranthus tuberculatus* accessions from Wisconsin at 21 days after treatment with 2,4‐D, atrazine, glyphosate, fomesafen, and mesotrione herbicides post‐emergence

Herbicide	Accession	ED_50_ percent biomass^†^ (g ai or ae ha^−1^)	ED_50_ RI^‡^	ED_50_ RI *P* value
2,4‐D	A75	126.7 (±13.0)	1.3	0.0712
	A101	299.9 (±29.0)	3.2	< 0.0001
	A103	59.3 (±8.1)	0.6	0.0005
	A82	94.1 (±9.3)	—^§^	—
Atrazine	A75	860.2 (±203.2)	5.6	0.0074
	A101	426.3 (±81.1)	2.8	0.0185
	A103	1888.0 (±441.3)	12.4	0.0026
	A82	152.8 (±29.5)	—	—
Glyphosate	A75	3221.0 (±799.6)	10.3	0.0017
	A101	2191.4 (±420.6)	7.0	0.0004
	A103	816.1 (±167.6)	2.6	0.0140
	A82	313.4 (±44.4)	—	—
Fomesafen	A75	7.9 (±2.0)	—	0.3591
	A101	47.0 (±7.4)	8.5	0.0013
	A103	13.0 (±2.5)	2.4	0.0531
	A82	5.5 (±1.2)	—	—
Mesotrione	A75	6.2 (±0.7)	—	0.8800
	A101	11.3 (±1.1)	1.8	0.0086
	A103	6.2 (±0.8)	—	0.9255
	A82	6.3 (±0.8)	—	—

^†^ ED_50_ percent biomass, the estimated herbicide rate that decreased percent biomass by 50% compared with the respective herbicide non‐treated control.

^‡^ ED_50_ RI, for each herbicide, the resistance index (RI) was calculated by dividing the ED_50_ of the respective accession by the ED_50_ of the susceptible accession (A82).

^§^ —, the ED_50_ RI and ED_50_ RI *P* value were not calculated for the susceptible accession (A82) or when ED_50_ RI *P* value was not significant by Student's *t*‐test (*α* = 0.1).

Standard errors are shown in parentheses.

**Figure 1:**
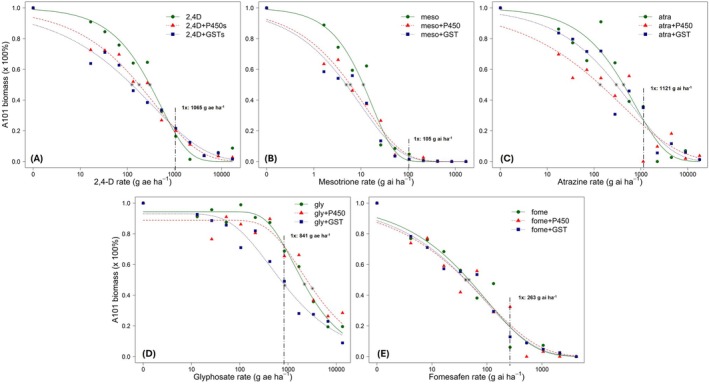
Dose–response curves comparing percent biomass of the multiple metabolic herbicide‐resistant (A101) *Amaranthus tuberculatus* accession from Wisconsin at 21 days after treatment with 2,4‐D (A), mesotrione (B), atrazine (C), glyphosate (D), and fomesafen (E) applied post‐emergence. Each herbicide was evaluated under three conditions: without pre‐treatment (green), herbicide applied after pre‐treatment with a P450 inhibitor (malathion; herbicide + P450; red), and herbicide applied after pre‐treatment with a glutathione *S*‐transferase (GST) inhibitor (4‐chloro‐7‐nitrobenzofurazan; herbicide + GST; blue). Vertical dash–dot lines indicate the respective 1× herbicide label rate. Asterisks indicate the ED_50_ percent biomass (estimated herbicide rate that decreased percent biomass by 50% compared with the respective herbicide non‐treated control).

The A101 accession exhibited an ED_50_ of 299.9 g ae ha^−1^ for 2,4‐D (RI = 3.2, *P* < 0.0001) (Table 4), an ED_50_ of 426.3 g ai ha^−1^ for atrazine (RI = 2.8, *P* = 0.0185), an ED_50_ of 2191.4 g ae ha^−1^ for glyphosate (RI = 7.0, *P* = 0.0004), an ED_50_ of 47.0 g ai ha^−1^ for fomesafen (RI = 8.5, *P* = 0.0013), and an ED_50_ of 11.3 g ai ha^−1^ for mesotrione (RI = 1.8, *P* = 0.0086). For A101, P450s and GSTs inhibition reduced 2,4‐D ED_50_ by 41% (177.7 g ae ha^−1^, *P* < 0.0001) (Table 2 and Fig. 1(A); Supporting Information, Fig. S1) and 58% (124.8 g ae ha^−1^, *P* <0.0001), respectively, compared with 2,4‐D alone. Similarly, P450s and GSTs inhibition reduced mesotrione ED_50_ by 47% (6.0 g ai ha^−1^, *P* <0.0001) (Table 2 and Fig. 1(B); Supporting Information, Fig. S2) and 57% (4.9 g ai ha^−1^, *P* <0.0001), respectively, compared with mesotrione alone. The inhibition of P450s reduced atrazine ED_50_ by 68% (134.8 g ai ha^−1^, *P* <0.0001) (Table 2 and Fig. 1(C); Supporting Information, Fig. S3) compared with atrazine alone. The inhibition of GSTs reduced glyphosate ED_50_ by 61% (856.7 g ae ha^−1^, *P* <0.0001) (Table 2 and Fig. 1(D); Supporting Information, Fig. S4) compared with glyphosate alone. The inhibition of P450s or GSTs did not change fomesafen ED_50_ for A101 (Table 2 and Fig. 1(E); Supporting Information, Fig. S5).

The A103 accession exhibited an ED_50_ of 1888.0 g ai ha^−1^ for atrazine (RI = 12.4, *P* = 0.0026) (Table 4), an ED_50_ of 816.1 g ae ha^−1^ for glyphosate (RI = 2.6, *P* = 0.0140), and an ED_50_ of 13.0 g ai ha^−1^ for fomesafen (RI = 2.4, *P* = 0.0531). For A103, P450s inhibition reduced atrazine ED_50_ by 63% (704.8 g ai ha^−1^, *P* <0.0001) (Table 2) compared with atrazine alone. Conversely, P450s and GSTs inhibition increased fomesafen ED_50_ by 108% (27.1 g ai ha^−1^, *P* = 0.0234) (Table 2) and 147% (32.1 g ai ha^−1^, *P* = 0.0155), respectively, compared with fomesafen alone. The inhibition of P450s or GSTs did not change glyphosate ED_50_ for A103 (Table 2).

For A82 (susceptible), P450s inhibition reduced atrazine ED_50_ and mesotrione ED_50_ by 31% (104.9 g ai ha^−1^, *P* = 0.0522) (Table 2) and 32% (4.3 g ai ha^−1^, *P* = 0.0156), respectively, compared with atrazine and mesotrione alone, respectively. The inhibition of P450s or GSTs did not change 2,4‐D, glyphosate, and fomesafen ED_50_ for A82 (data not shown).

### 3.2 Assessment of ametryne resistance in *A. tuberculatus* accessions

**Figure 2:**
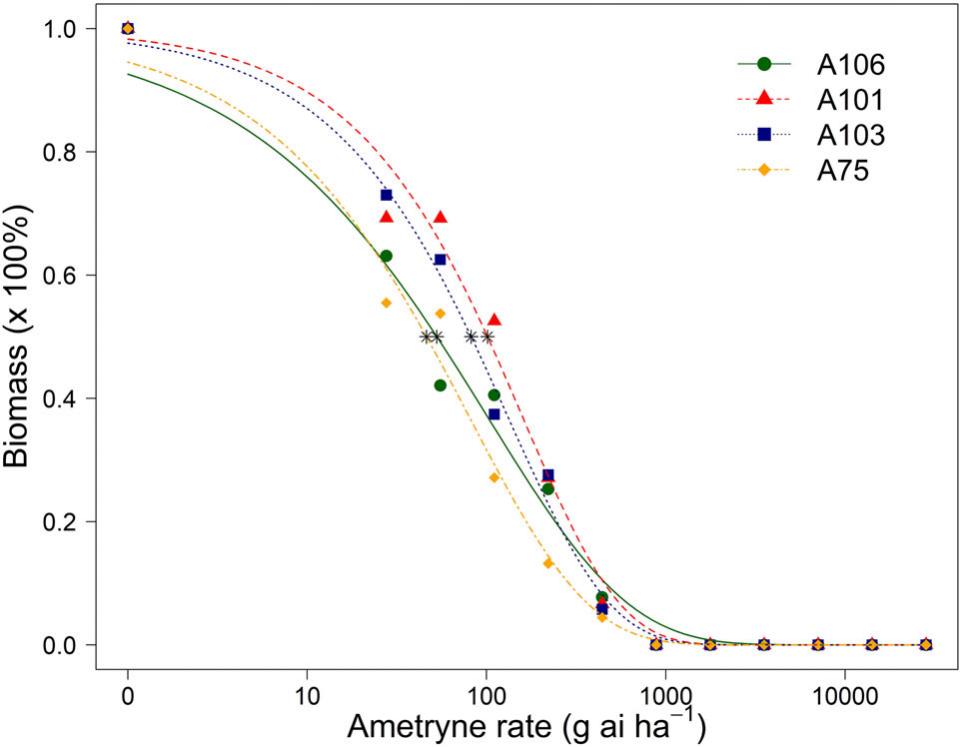
Dose–response curves comparing percent biomass of the A75, A101, and A103 accessions (multiple herbicide resistant) and the A106 accession (susceptible) of *Amaranthus tuberculatus* from Wisconsin at 21 days after treatment with ametryne applied postemergence. Vertical dash–dot lines indicate the respective 1× herbicide label rate. Asterisks indicate the ED_50_ percent biomass (estimated herbicide rate that decreased percent biomass by 50% compared with the respective herbicide non‐treated control).

Compared with A106 (susceptible), the A101 and A103 accessions exhibited a low‐level resistance to ametryne (ED_50_ = 101.3 and 82.1 g ai ha^−1^, respectively; RI = 1.9 and 1.6, respectively; *P* = 0.0111 and 0.0620, respectively) (Table 5 and Fig. 2), whereas the A75 accession was susceptible (*P* = 0.5583).

**Table 5:** Dose–response model output comparing the A75, A101, and A103 (multiple herbicide resistant) and A106 (susceptible) *Amaranthus tuberculatus* accessions from Wisconsin at 21 days after treatment with ametryne POST

Herbicide	Accession	ED_50_ percent biomass^†^	ED_50_ RI^‡^	ED_50_ RI *P*‐value
		(g ai ha^−1^)		
Ametryne	A75	46.3 (±6.7)	—^§^	0.5583
	A101	101.3 (±10.4)	1.9	0.0111
	A103	82.1 (±8.8)	1.6	0.0620
	A106	52.9 (±8.3)	—	—

^†^ ED_50_ percent biomass, the estimated herbicide rate that decreased percent biomass by 50% compared with the respective non‐treated control.

^‡^ ED_50_ RI, the resistance index (RI) was calculated by dividing the ED_50_ of the respective accession by the ED_50_ of the susceptible accession (A106).

^§^ —, the ED_50_ RI and ED_50_ RI *P* value were not calculated for the susceptible accession (A106) or when ED_50_ RI *P* value was not significant by Student's *t*‐test (*α* = 0.1).

Standard errors are shown in parentheses.

### 3.3 Assessment of target‐site herbicide resistance in *A. tuberculatus* accessions

The S264G substitution in the *psbA* gene was absent in all four accessions evaluated (Table 3). The A101, A103, and A106 (susceptible) accessions exhibited segregating variation for P106S in the *EPSPS* gene, with a frequency of resistant alleles ranging from 0 to 0.174 for A101 (Table 3; Supporting Information, Fig. S6), from 0.140 to 0.305 for A103, and from 0 to 0.255 for A106. Moreover, A75, A101, and A103 accessions exhibited enhanced *EPSPS* expression, with a FC of up to 3.41, 2.16, and 1.92, respectively, compared with the control group in the DGE analysis (Table 6). The A101, A103, and A106 (susceptible) accessions exhibited segregating variation for ΔG210 in *PPX2*, with a frequency of resistant alleles ranging from 0.291 to 0.364 for A101 (Table 3; Supporting Information, Fig. S6), from 0.124 to 0.275 for A103, and from 0 to 0.423 for A106. The *PPX2* R128G substitution was not present in any of the accessions evaluated.

**Table 6:** Differential gene expression analysis exhibiting changes in the expression of herbicide target genes *psbA*, *EPSPS*, *PPX1*, and *PPX2* in *Amaranthus tuberculatus* accessions. Pools A106_P1, A92_P1, A92_P2, and A92_P3 were used to compose the control group for comparisons

Gene	Gene ID	Accession	*P*‐value	FDR^†^	FC^†^
*psbA*	AmaTu_RefChr00g253270	A75	0.2099	0.3512	−1.56
		A101	0.2538	0.4240	1.47
		A103	0.1323	0.3562	−1.72
*EPSPS*	AmaTu_RefChr06g108660	A75	< 0.0001	0.0007	3.41
		A101	0.0002	0.0055	2.16
		A103	0.0010	0.0272	1.92
*PPX1*	AmaTu_RefChr13g204850	A75	0.0001	0.0037	−1.51
		A101	0.0066	0.0385	−1.28
		A103	0.0035	0.0531	−1.31
*PPX2*	AmaTu_RefChr07g123900	A75	0.2972	0.4467	−1.08
		A101	0.0224	0.0852	−1.21
		A103	0.5310	0.7404	−1.05

^†^ FDR, false discovery rate; FC, fold‐change.

## 4 DISCUSSION

Since 2009 *A. tuberculatus* populations resistant to 2,4‐D have been reported in Nebraska, Illinois, Missouri, and Wisconsin,^6^, ^54^, ^55^, ^56^ with enhanced metabolism, likely mediated by P450s, underlying resistance to 2,4‐D in the Nebraska, Illinois, and Missouri populations.^17^, ^18^, ^19^, ^20^ Further research confirmed that the Illinois population is also resistant to dicamba,^57^ likely because of an enhanced oxidative stress tolerance and potential conjugation via GSTs of dicamba and its by‐products.^26^ More recent genetic studies demonstrated that resistance to 2,4‐D and dicamba is controlled by distinct loci in this Illinois population.^58^ Moreover, unpublished results identified 2,4‐D metabolites, suggesting the involvement of P450s, GSTs, and glucosyltransferases in 2,4‐D metabolic resistance.^59^ Similarly, our results using P450‐ and GST‐inhibitor assays suggest that both enzyme groups contribute to 2,4‐D resistance in the A101 accession, indicating a potential metabolic basis similar to that observed in other resistant populations. However, pre‐treatment with either inhibitor did not alter the response of accession A75 to 2,4‐D.

Atrazine resistance in *A. tuberculatus* has been reported to be conferred by both TSR and NTSR mechanisms. TSR to atrazine is typically conferred by a point mutation in the *psbA* gene encoding the PSII D1 target protein, which results in a substitution of serine to glycine at amino acid position 264 that significantly reduces atrazine binding.^42^ In addition, rapid detoxification of atrazine through GSTs has been reported in various *A. tuberculatus* populations.^4^, ^25^, ^27^, ^28^, ^31^, ^60^, ^61^ In two previous studies that evaluated the response of atrazine‐resistant *A. tuberculatus* populations to a pre‐treatment with the P450 inhibitor malathion followed by atrazine application, the pre‐treatment did not alter resistance, suggesting that P450s were likely not involved with atrazine resistance in their populations.^20^, ^25^ However, in species such as annual ryegrass (*Lolium rigidum* Gaud.), P450s were shown to be associated with PSII‐inhibitor resistance, including atrazine.^62^, ^63^, ^64^ In our study, the S264G substitution was absent in A75, A101, A103, and A106 (susceptible) accessions. According to our results, both P450s and GSTs appear to be associated with atrazine resistance in A75, which was susceptible to ametryne. By contrast, atrazine resistance in A101 and A103 accessions, which also exhibited a low‐level resistance to ametryne, seem to be associated with P450s. Moreover, P450 seems to be associated with atrazine tolerance enhancement in A82 (susceptible accession).

Three glyphosate‐resistance mechanisms have been reported in *A. tuberculatus*, including the target‐site substitution P106S, enhanced expression of the *EPSPS* gene, and reduced glyphosate translocation.^4^, ^43^, ^65^, ^66^ Moreover, glyphosate‐resistant Palmer amaranth (*Amaranthus palmeri* S. Watson) populations with increased *EPSPS* copy number also exhibited greater reactive oxygen species (ROS) scavenging activity associated with elevated levels of antioxidant metabolites derived from the phenylpropanoid pathway. Consequently, increased oxidant quenching efficiency could potentially complement glyphosate resistance in these populations.^67^ In multiple species, GSTs are reported to be involved in regulating the accumulation of various antioxidant secondary metabolites derived from the phenylpropanoid pathway, including anthocyanins and flavonoids.^12^ Thale cress, *Arabidopsis thaliana* (L.) Heynh., plants overexpressing *AmGSTF1* from multiple herbicide‐resistant blackgrass (*Alopecurus myosuroides* Huds.) exhibited increased resistance to multiple herbicides that were associated with overaccumulation of protective flavonoids.^68^ In this context, our results suggest that GSTs may be indirectly associated with glyphosate detoxification in A101 by reducing the effect of oxidative stress caused by herbicide‐induced ROS.

Moreover, our results suggest a segregating variation for the P106S mutation in *EPSPS* in accessions A101 and A103. This mutation was also detected in the A106 (susceptible) accession, because resistance may be segregating at low frequency. Differential expression analysis of RNA sequencing data indicated that accessions A75, A101, and A103 exhibited modest *EPSPS* overexpression (1.92‐ to 3.41‐fold). Previous work in kochia, *Bassia scoparia* (L.) A. J. Scott, reported a linear relationship between *EPSPS* gene copy number and *EPSPS* transcript abundance of ~2.7:1,^69^ suggesting that increased EPSPS transcription can be associated with *EPSPS* gene amplification. Although this relationship has not been confirmed in *A. tuberculatus*, the *EPSPS* overexpression observed here is consistent with modest *EPSPS* amplification. The number of *EPSPS* copies required to confer meaningful resistance varies among species. For example, in *B. scoparia*, individuals with fewer than three *EPSPS* copies did not exhibit noticeable resistance under field conditions.^70^ In *A. palmeri*, glyphosate resistance increased significantly up to ~15 *EPSPS* copies, but additional copies beyond this threshold resulted in only small increases in resistance.^71^ Although *EPSPS* upregulation was relatively low in the accessions from our study, the combination of segregating TSR (P106S) and moderately enhanced *EPSPS* expression may contribute to the low to moderate glyphosate resistance observed in A75, A101, and A103. Likewise, because resistance mechanisms were assessed using pooled samples in this study, it is also possible that copy number variation and/or TSR alleles were not fixed within populations, such that population‐level expression observed may reflect mixtures of low‐copy and high‐copy individuals.

*Amaranthus tuberculatus* was the first weed species to evolve resistance to PPO inhibitors,^4^, ^72^ with now both ΔG210 deletion and R128G/I substitution conferring TSR.^6^, ^44^, ^45^, ^55^ Moreover, enhanced herbicide detoxification via P450s and GSTs has also been reported to be associated with NTSR in *A. tuberculatus* and *A. palmeri*.^21^, ^73^, ^74^, ^75^ Recently, in Wisconsin, an *A. tuberculatus* population (A92 accession) was identified that was resistant to PPO inhibitors applied either pre‐emergence or post‐emergence, without the presence of any known target‐site mutations in the *PPX1* or *PPX2* genes and likely with a NTSR mechanism conferring this resistance.^5^ In our study, A101, A103, and A106 (susceptible) accessions segregated for ΔG210; the R128G was not present in any of the accessions evaluated. According to our results, neither P450s nor GSTs are associated with fomesafen resistance in A101 and A103 accessions. Because A106 is a glasshouse‐grown F_1_ generation of A82 parent plants, which were collected from the field without generating plants homozygous for susceptibility, we believe this may be the reason why A106 is segregating for ΔG210.

Resistance to HPPD inhibitors is still considered rare, with only five weed species reported to have evolved resistance to date, namely *A. tuberculatus*, *A. palmeri*, redroot pigweed (*Amaranthus retroflexus* L.), wild radish (*Raphanus raphanistrum* L.), and Chinese sprangletop (*Leptochloa chinensis* (L.) Nees).^3^, ^76^, ^77^, ^78^ Several studies have indicated the role of P450s in HPPD resistance in *A. tuberculatus*.^4^, ^23^, ^24^, ^25^ Moreover, in four of the five weed species that have evolved HPPD resistance, including *A. tuberculatus*, detoxification of HPPD inhibitors was reported to occur through multiple pathways, likely catalyzed by P450s, aldo‐keto reductase enzymes, GSTs, and other non‐P450s oxygenases.^29^, ^77^, ^78^, ^79^ Similarly, our results demonstrated that P450s and GSTs may be associated with the low‐level resistance to mesotrione in the A101 accession, whereas P450s are associated with mesotrione tolerance enhancement in A82 (susceptible accession).

## 5 CONCLUSIONS

In conclusion, our results suggest that A75 is resistant to 2,4‐D (low‐level resistance; unknown mechanism), atrazine (P450s and GSTs), and glyphosate (enhanced *EPSPS* expression). The A101 accession is resistant to 2,4‐D (P450s and GSTs), mesotrione (low‐level resistance; P450s and GSTs), atrazine (P450s), glyphosate (GSTs, P106S and enhanced *EPSPS* expression), and fomesafen (ΔG210). The A103 accession is resistant to atrazine (P450s), glyphosate (P106S and enhanced *EPSPS* expression), and fomesafen (*Δ*G210). To our knowledge, this is the first report of P450s associated with atrazine resistance in *A. tuberculatus* globally. The S264G substitution, known for conferring atrazine resistance, and R128G substitution, known for conferring fomesafen resistance, were absent in all four *A. tuberculatus* accessions evaluated.

The A101 accession was sampled from a field with no history of atrazine use or exposure of *A. tuberculatus* plants to 2,4‐D, but it was still resistant to these two herbicides. This suggests that the use of other herbicides may have contributed to the selection of enhanced P450s and GSTs activity in this population. Moreover, the A101 accession is the first confirmed case of *A. tuberculatus* resistance to HPPD inhibitors in Wisconsin, exhibiting a low‐level resistance likely associated with both P450s‐ and GSTs‐mediated metabolism. In addition, our results suggest the coexistence of TSR and NTSR mechanisms associated with glyphosate resistance in this accession. Further research is necessary to elucidate the 2,4‐D low‐level resistance mechanism in the A75 accession. Finally, our findings highlight the complexity of herbicide resistance and emphasize the importance of integrated weed management, including cultural and physical control, smart technologies, novel approaches for herbicide and bioherbicide discovery, community efforts and policies.^80^, ^81^, ^82^, ^83^, ^84^, ^85^, ^86^, ^87^, ^88^

## CONFLICT OF INTEREST

The authors declare no conflicts of interest.

## Supporting information


**Table S1.** Additional mutation sites in the *psbA, PPX1, PPX2*, and *EPSPS* genes examined in our study which are associated with herbicide resistance in multiple weed species other than *Amaranthus tuberculatus*.
**Fig. S1.** Plants of the susceptible (A82) and multiple metabolic herbicide‐resistant (A101) *Amaranthus tuberculatus* accession from Wisconsin at 21 days after treatment with 2,4‐D; 2,4‐D + P450‐inhibitor; and 2,4‐D + GST‐inhibitor. Herbicide rates ranged from 0x to 16x the label rate of 2,4‐D (Table 1), with rates increasing from the left to the right side within the picture in a zig‐zag way. The P450‐ and GST‐inhibitor, and adjuvants rates were maintained at 1x (Table 1).
**Fig. S2.** Plants of the susceptible (A82) and multiple metabolic herbicide‐resistant (A101) *Amaranthus tuberculatus* accession from Wisconsin at 21 days after treatment with mesotrione; mesotrione+P450‐inhibitor; and mesotrione+GST‐inhibitor. Herbicide rates ranged from 0x to 16x the label rate of mesotrione (Table 1), with rates increasing from the left to the right side within the picture in a zig‐zag way (rates up to 2x are shown). The P450‐ and GST‐inhibitor, and adjuvants rates were maintained at 1x (Table 1).
**Fig. S3.** Plants of the susceptible (A82) and multiple metabolic herbicide‐resistant (A101) *Amaranthus tuberculatus* accession from Wisconsin at 21 days after treatment with atrazine; atrazine +P450‐inhibitor; and atrazine +GST‐inhibitor. Herbicide rates ranged from 0x to 16x the label rate of atrazine (Table 1), with rates increasing from the left to the right side within the picture in a zig‐zag way (rates up to 2x are shown). The P450‐ and GST‐inhibitor, and adjuvants rates were maintained at 1x (Table 1).
**Fig. S4.** Plants of the susceptible (A82) and multiple metabolic herbicide‐resistant (A101) *Amaranthus tuberculatus* accession from Wisconsin at 21 days after treatment with glyphosate; glyphosate +P450‐inhibitor; and glyphosate +GST‐inhibitor. Herbicide rates ranged from 0x to 16x the label rate of glyphosate (Table 1), with rates increasing from the left to the right side within the picture in a zig‐zag way (rates up to 4x are shown). The P450‐ and GST‐inhibitor, and adjuvants rates were maintained at 1x (Table 1).
**Fig. S5.** Plants of the susceptible (A82) and multiple metabolic herbicide‐resistant (A101) *Amaranthus tuberculatus* accession from Wisconsin at 21 days after treatment with fomesafen; fomesafen +P450‐inhibitor; and fomesafen +GST‐inhibitor. Herbicide rates ranged from 0x to 16x the label rate of fomesafen (Table 1), with rates increasing from the left to the right side within the picture in a zig‐zag way (rates up to 4x are shown). The P450‐ and GST‐inhibitor, and adjuvants rates were maintained at 1x (Table 1).
**Fig. S6.** Transcriptome coverage plots across loci surrounding (A) *PPX2* ΔG210 and (B) *EPSPS* P106S residues involved in target‐site resistance to PPO‐ and EPSPS‐inhibitors, respectively. Bases at the site of ΔG210 deletion and P106S substitution are indicated by a red bar at the top and variants are colored by base according to their frequency in each pool.

## Data Availability

The data that support the findings of this study are available from the corresponding author upon reasonable request.
