# Supplementary material for: Target‐site and non‐target‐site mechanisms confer multiple herbicide resistance in waterhemp ( Amaranthus tuberculatus ) accessions from Wisconsin
Source: Pest Manag Sci. 2026 Mar 2;82(6):5695–706. doi: 10.1002/ps.70672 (PMC13158449; doi:10.1002/ps.70672)
Supplement: Supplementary file 1 — Table S1. Additional mutation sites in the psbA, PPX1, PPX2, and EPSPS genes examined in our study which are associated with herbicide resistance in multiple weed species other than Amaranthus tuberculatus. Fig. S1. Plants of the susceptible (A82) and multiple metabolic herbicide‐resistant (A101) Amaranthus tuberculatus accession from Wisconsin at 21 days after treatment with 2,4‐D; 2,4‐D + P450‐inhibitor; and 2,4‐D + GST‐inhibitor. Herbicide rates ranged from 0x to 16x the label rate of 2,4‐D (Table 1), with rates increasing from the left to the right side within the picture in a zig‐zag way. The P450‐ and GST‐inhibitor, and adjuvants rates were maintained at 1x (Table 1). Fig. S2. Plants of the susceptible (A82) and multiple metabolic herbicide‐resistant (A101) Amaranthus tuberculatus accession from Wisconsin at 21 days after treatment with mesotrione; mesotrione+P450‐inhibitor; and mesotrione+GST‐inhibitor. Herbicide rates ranged from 0x to 16x the label rate of mesotrione (Table 1), with rates increasing from the left to the right side within the picture in a zig‐zag way (rates up to 2x are shown). The P450‐ and GST‐inhibitor, and adjuvants rates were maintained at 1x (Table 1). Fig. S3. Plants of the susceptible (A82) and multiple metabolic herbicide‐resistant (A101) Amaranthus tuberculatus accession from Wisconsin at 21 days after treatment with atrazine; atrazine +P450‐inhibitor; and atrazine +GST‐inhibitor. Herbicide rates ranged from 0x to 16x the label rate of atrazine (Table 1), with rates increasing from the left to the right side within the picture in a zig‐zag way (rates up to 2x are shown). The P450‐ and GST‐inhibitor, and adjuvants rates were maintained at 1x (Table 1). Fig. S4. Plants of the susceptible (A82) and multiple metabolic herbicide‐resistant (A101) Amaranthus tuberculatus accession from Wisconsin at 21 days after treatment with glyphosate; glyphosate +P450‐inhibitor; and glyphosate +GST‐inhibitor. Herbicide rates ranged from 0x to 16x the [file PS-82-5695-s001.docx]

**Supporting Information**

**Table S1.** Additional mutation sites in the *psbA, PPX1, PPX2,* and *EPSPS* genes examined in our study which are associated with herbicide resistance in multiple weed species other than *Amaranthus tuberculatus.*

| Mutation site | Gene target | WSSA SOA^†^ | Resistant allele | Weed species | Reference |
| --- | --- | --- | --- | --- | --- |
|  |  |  |  |  |  |
| Val-219-Ile | *psbA* | PSII (5) | Valine to Isoleucine | *Poa annua* | Mengistu et al. 2000 |
| Ala-251-Val | *psbA* | PSII (5) | Alanine to Valine | *Chenopodium album* | Mechant et al. 2008 |
| Phe-255-Ile | *psbA* | PSII (5) | Phenylalanine to Isoleucine | *Capsella bursa-pastoris* | Perez-Jones et al. 2009 |
| Asn-266-Thr | *psbA* | PSII (5) | Asparagine to Threonine | *Senecio vulgaris* | Park and Mallory-Smith 2006 |
| Phe-274-Val | *psbA* | PSII (5) | Phenylalanine to Valine | *Raphanus raphanistrum* | Lu et al. 2019 |
| Thr-102-Ile | *EPSPS* | EPSPS (9) | Threonine to Isoleucine | *Tridax procumbens* | Li et al. 2018 |
| Ala-103-Val | *EPSPS* | EPSPS (9) | Alanine to Valine | *Amaranthus hybridus* | Perotti et al. 2019 |
| Ala-212-Thr | *PPX1* | PPO (14) | Alanine to Threonine | *Eleusine indica* | Bi et al. 2020 |
| Val-361-Ala | *PPX2* | PPO (14) | Valine to Alanine | *Amaranthus palmeri* | Nie et al. 2023 |
| Gly-399- Ala | *PPX2* | PPO (14) | Glycine to Alanine | *Amaranthus palmeri* | Rangani et al. 2019 |

† Weed Science Society of America (WSSA) Herbicide Site of Action (SOA): PSII, photosynthesis at PSII - serine 264 binders (Group 5); EPSPS, enolpyruvyl shikimate phosphate synthase (Group 9); PPO, protoporphyrinogen oxidase (Group 14).

**References Table S1**

Bi B, Wang Q, Coleman JJ, Porri A, Peppers JM, Patel JD, Betz M, Lerchl J, McElroy JS, A novel mutation A212T in chloroplast Protoporphyrinogen oxidase (PPO1) confers resistance to PPO inhibitor Oxadiazon in *Eleusine indica*. Pest Manag Sci 76:1786-1794 (2020).

Li J, Peng Q, Han H, Nyporko A, Kulynych T, Yu Q, Powles S, Glyphosate resistance in *Tridax procumbens* via a novel EPSPS Thr-102-Ser substitution. J Agric Food Chem 66:7880-7888 (2018).

Lu H, Yu Q, Han H, Owen MJ, Powles SB, A novel psbA mutation (Phe274-Val) confers resistance to PSII herbicides in wild radish (*Raphanus raphanistrum*). Pest Manag Sci 75:144-151 (2019).

Mechant E, De Marez T, Hermann O, Olsson R, Bulcke R, Target site resistance to metamitron in *Chenopodium album* L. J Plant Dis Prot XXI:37-40 (2008).

Mengistu LW, Mueller-Warrant GW, Liston A, Barker E, psbA Mutation (valine_219_ to isoleucine) in *Poa annua* resistant to metribuzin and diuron. *Pest Manag Sci* 56:209-217 (2000).

Nie H, Harre NT, Young BG, A new v361a mutation in *Amaranthus palmeri PPX2* associated with PPO-inhibiting herbicide resistance. Plants 12:1886 (2023).

Park KW, Mallory-Smith CA, psbA mutation (Asn266 to Thr) in *Senecio vulgaris* L. confers resistance to several PS II-inhibiting herbicides. Pest Manag Sci 62:880-5 (2006).

Perez-Jones A, Intanon S, Mallory-Smith C, Molecular analysis of hexazinone-resistant shepherd’s-purse (*Capsella bursa-pastoris*) reveals a novel psbA mutation. Weed Sci 57:574-578 (2009).

Perotti VE, Larran AS, Palmieri VE, Martinatto AK, Alvarez CE, Tuesca D, Permingeat HR, A novel triple amino acid substitution in the EPSPS found in a high-level glyphosate-resistant *Amaranthus hybridus* population from Argentina. Pest Manag Sci 75:1242-1251 (2019).

Rangani G, Salas-Perez RA, Aponte RA, Knapp M, Craig IR, Mietzner T, Langaro AC, Noguera MM, Porri A, Roma-Burgos N, A novel single-site mutation in the catalytic domain of protoporphyrinogen oxidase ix (PPO) confers resistance to PPO-inhibiting herbicides. Front Plant Sci 10:568 (2019).


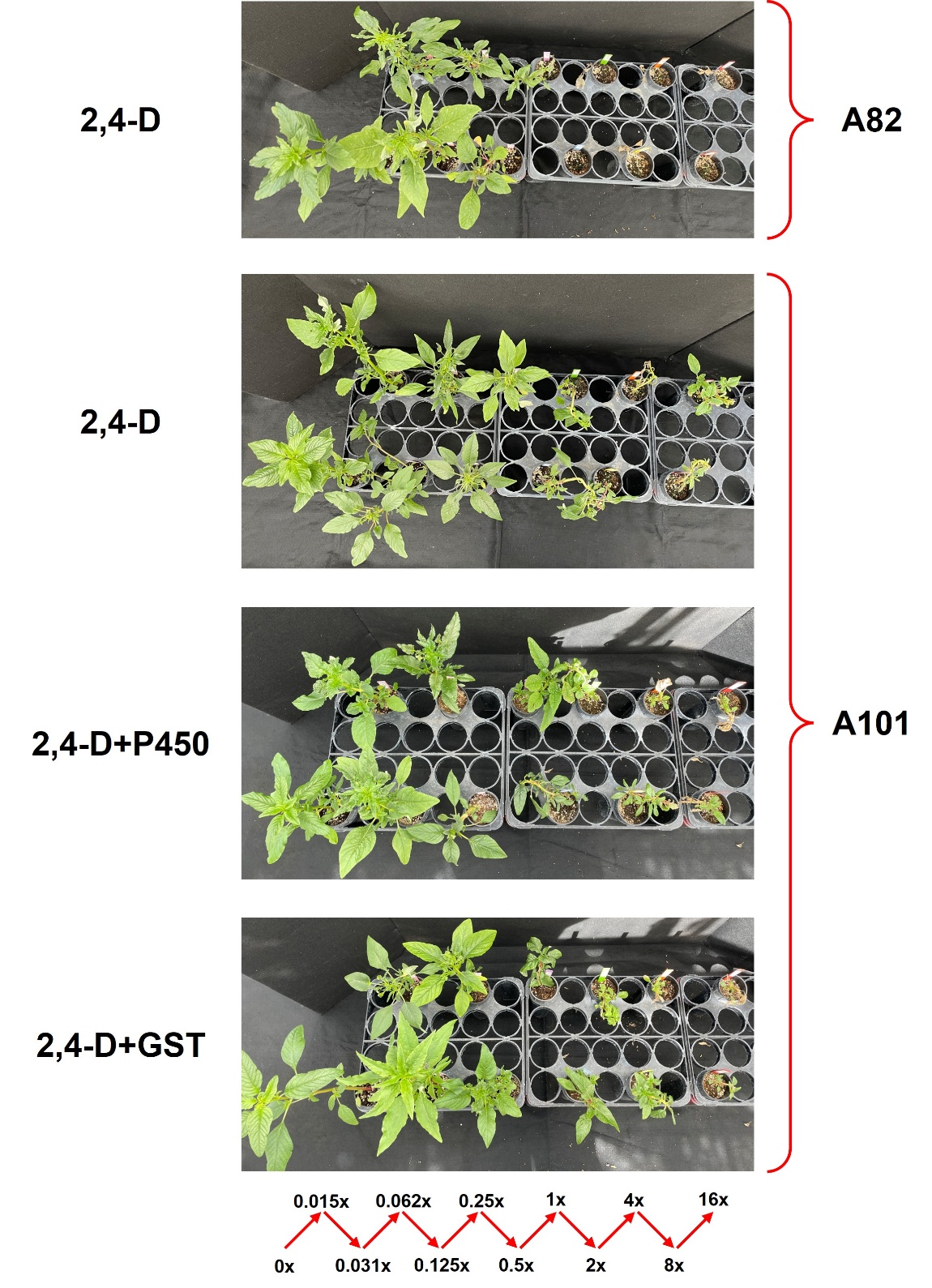


**Figure S1.** Plants of the susceptible (A82) and multiple metabolic herbicide-resistant (A101) *Amaranthus tuberculatus* accession from Wisconsin at 21 days after treatment with 2,4-D; 2,4-D+P450-inhibitor; and 2,4-D+GST-inhibitor. Herbicide rates ranged from 0x to 16x the label rate of 2,4-D (Table 1), with rates increasing from the left to the right side within the picture in a zig-zag way. The P450- and GST-inhibitor, and adjuvants rates were maintained at 1x (Table 1).


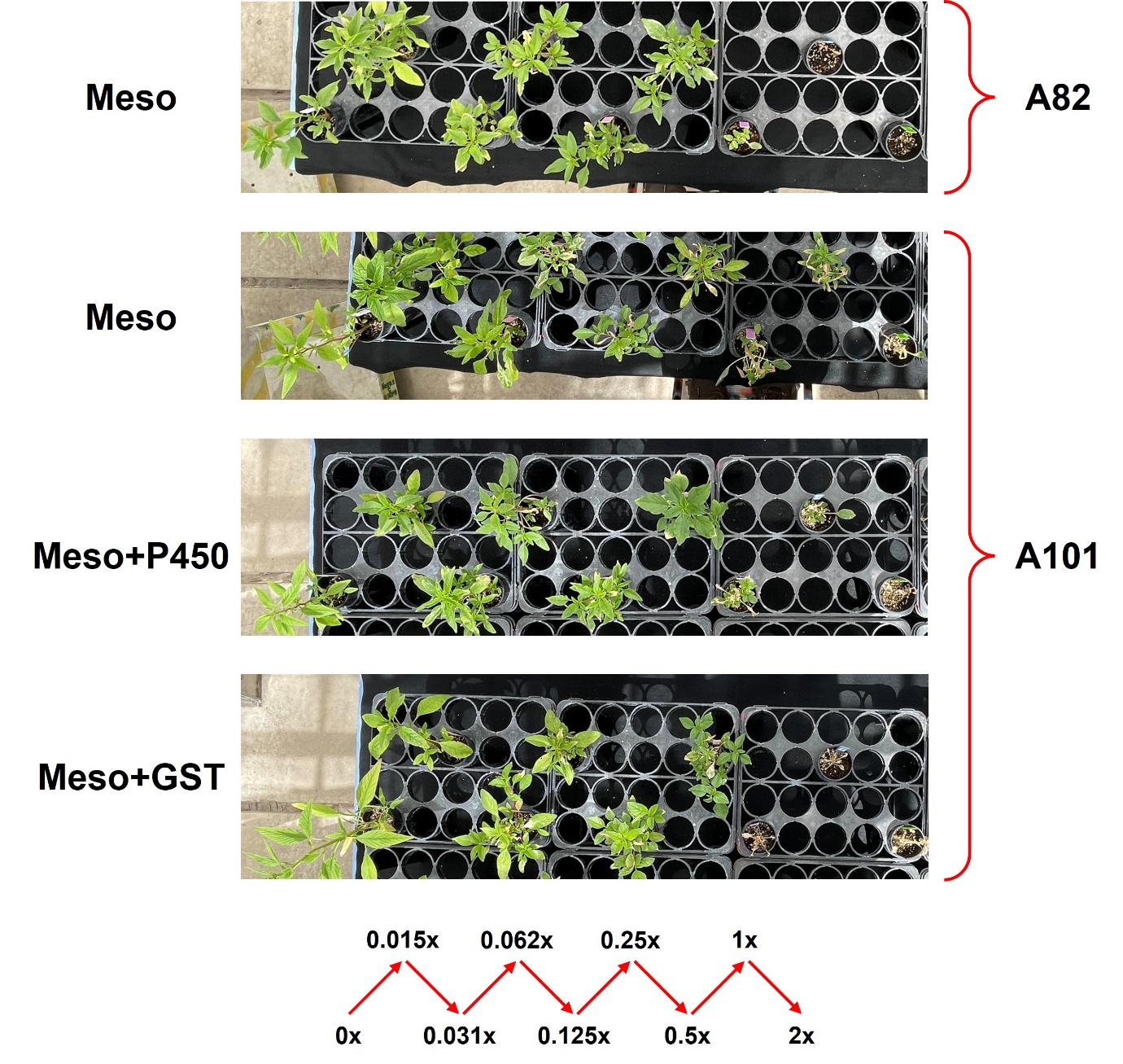


**Figure S2.** Plants of the susceptible (A82) and multiple metabolic herbicide-resistant (A101) *Amaranthus tuberculatus* accession from Wisconsin at 21 days after treatment with mesotrione; mesotrione+P450-inhibitor; and mesotrione+GST-inhibitor. Herbicide rates ranged from 0x to 16x the label rate of mesotrione (Table 1), with rates increasing from the left to the right side within the picture in a zig-zag way (rates up to 2x are shown). The P450- and GST-inhibitor, and adjuvants rates were maintained at 1x (Table 1).


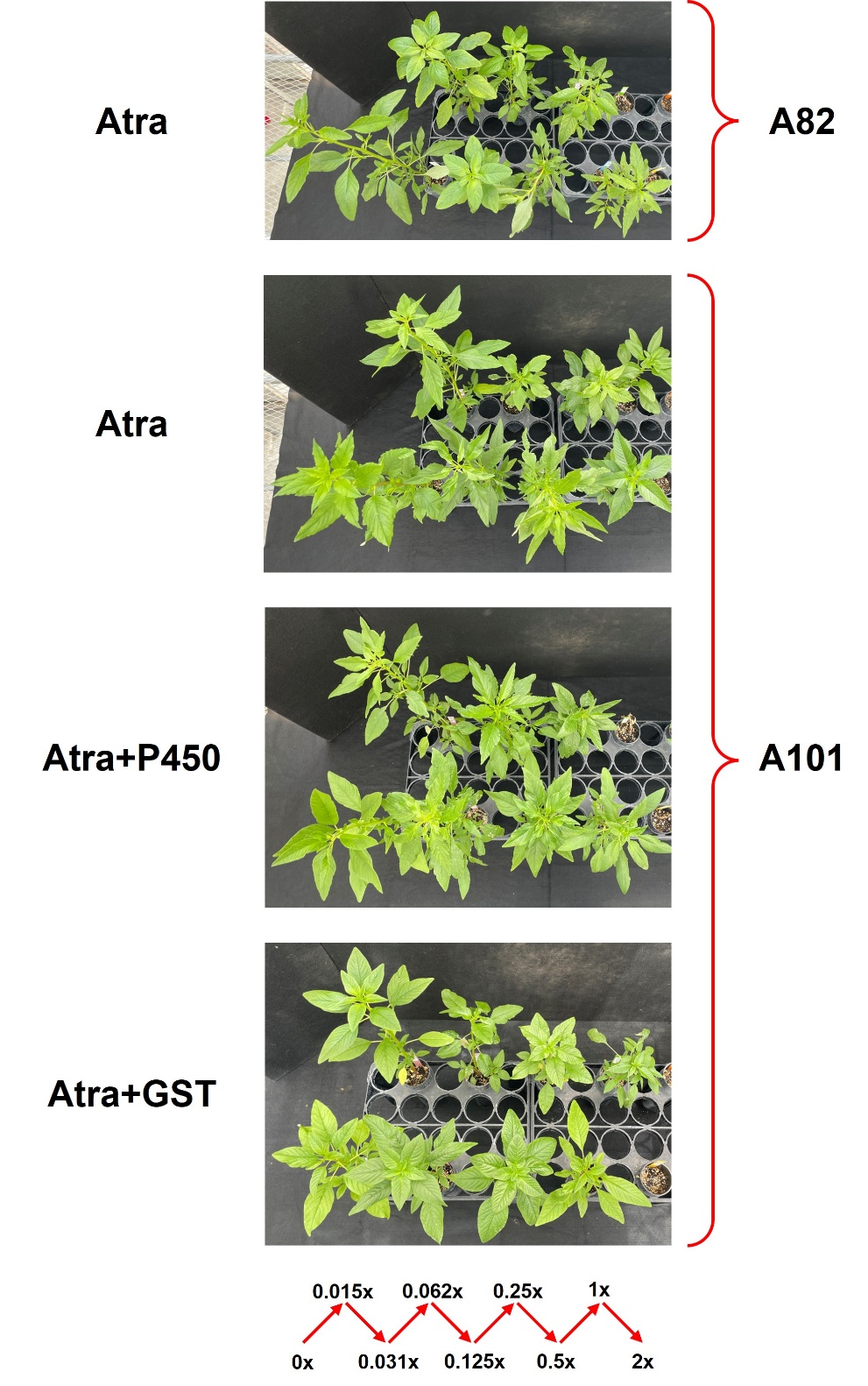


**Figure S3.** Plants of the susceptible (A82) and multiple metabolic herbicide-resistant (A101) *Amaranthus tuberculatus* accession from Wisconsin at 21 days after treatment with atrazine; atrazine +P450-inhibitor; and atrazine +GST-inhibitor. Herbicide rates ranged from 0x to 16x the label rate of atrazine (Table 1), with rates increasing from the left to the right side within the picture in a zig-zag way (rates up to 2x are shown). The P450- and GST-inhibitor, and adjuvants rates were maintained at 1x (Table 1).


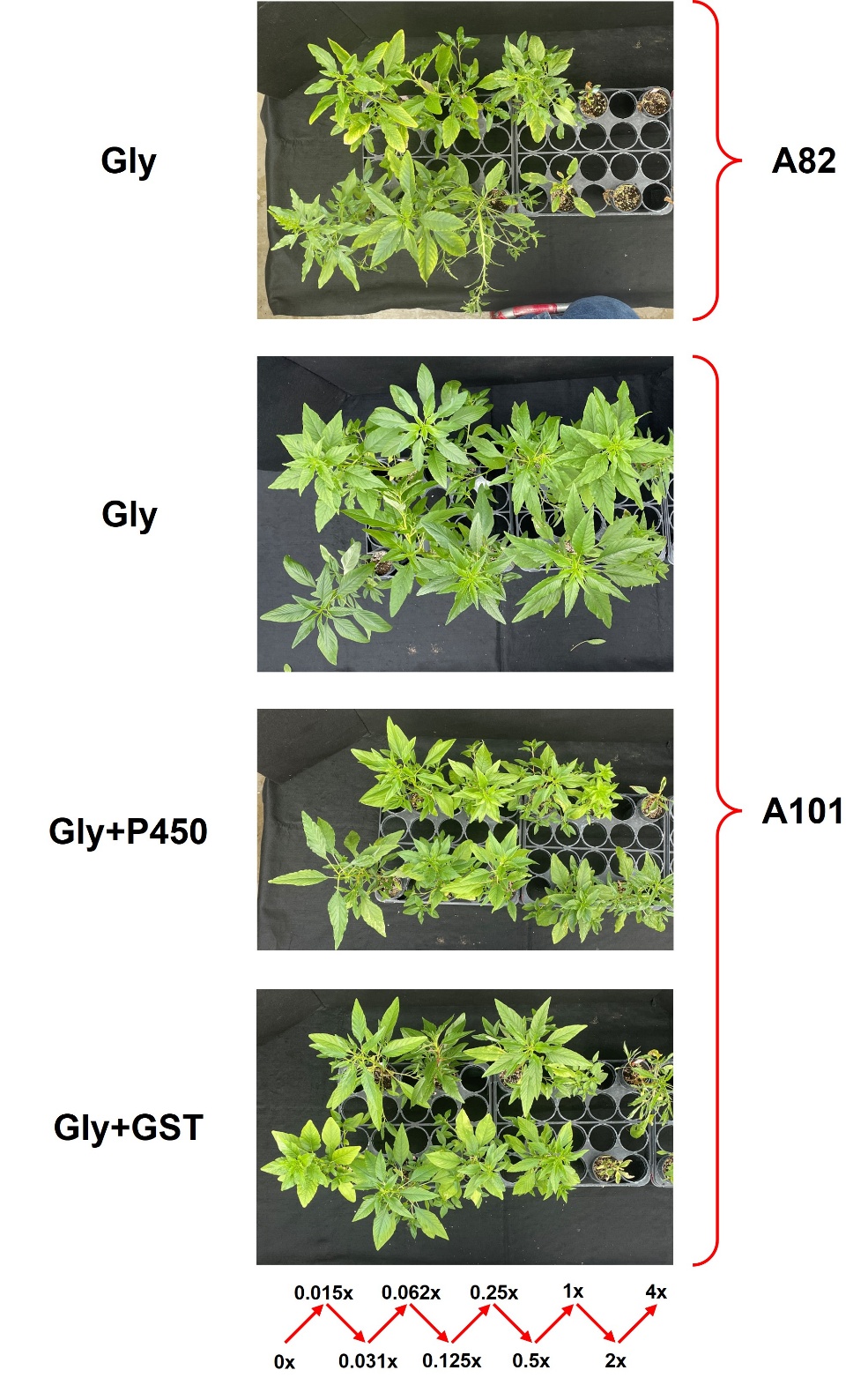


**Figure S4.** Plants of the susceptible (A82) and multiple metabolic herbicide-resistant (A101) *Amaranthus tuberculatus* accession from Wisconsin at 21 days after treatment with glyphosate; glyphosate +P450-inhibitor; and glyphosate +GST-inhibitor. Herbicide rates ranged from 0x to 16x the label rate of glyphosate (Table 1), with rates increasing from the left to the right side within the picture in a zig-zag way (rates up to 4x are shown). The P450- and GST-inhibitor, and adjuvants rates were maintained at 1x (Table 1).


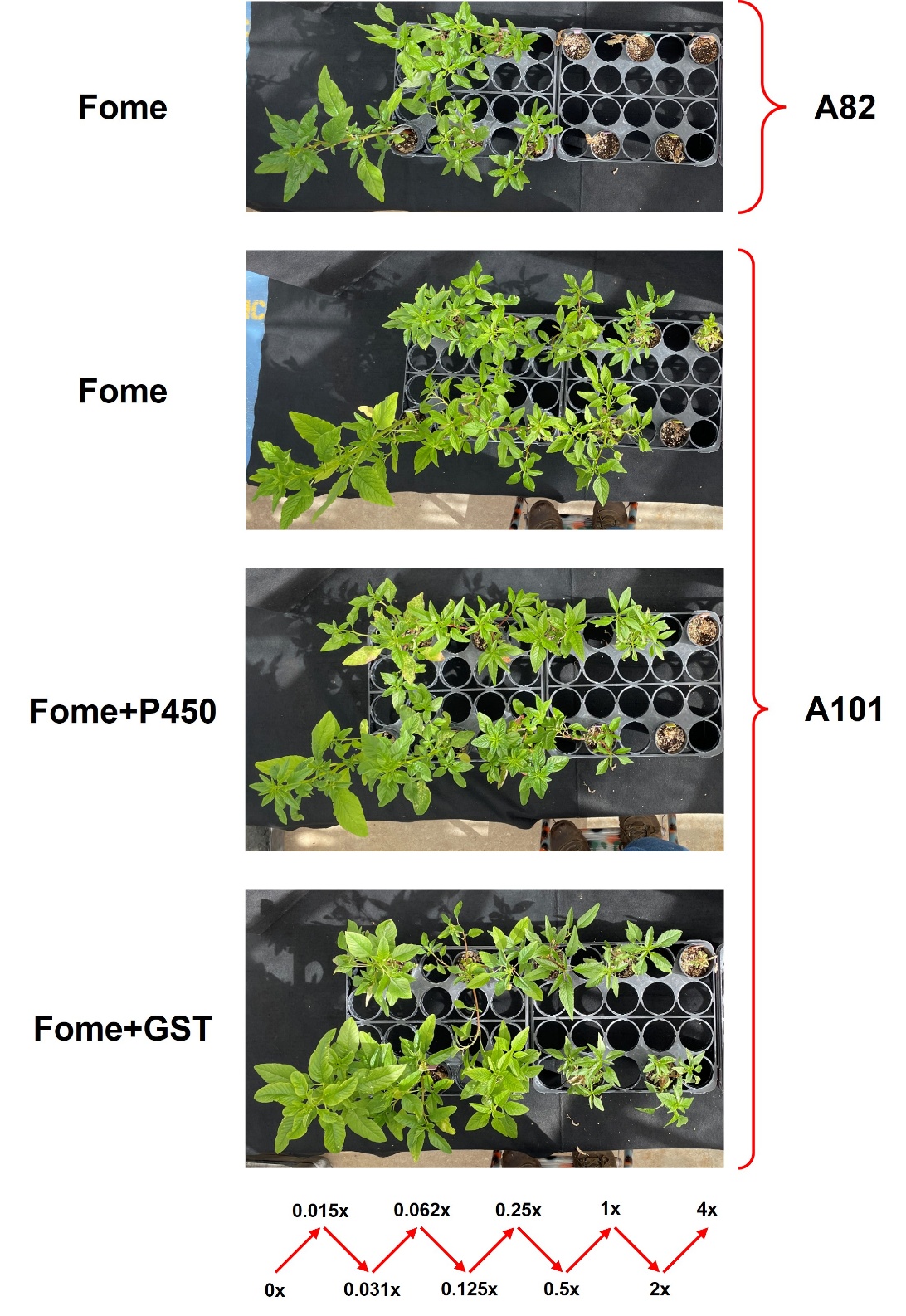


**Figure S5.** Plants of the susceptible (A82) and multiple metabolic herbicide-resistant (A101) *Amaranthus tuberculatus* accession from Wisconsin at 21 days after treatment with fomesafen; fomesafen +P450-inhibitor; and fomesafen +GST-inhibitor. Herbicide rates ranged from 0x to 16x the label rate of fomesafen (Table 1), with rates increasing from the left to the right side within the picture in a zig-zag way (rates up to 4x are shown). The P450- and GST-inhibitor, and adjuvants rates were maintained at 1x (Table 1).


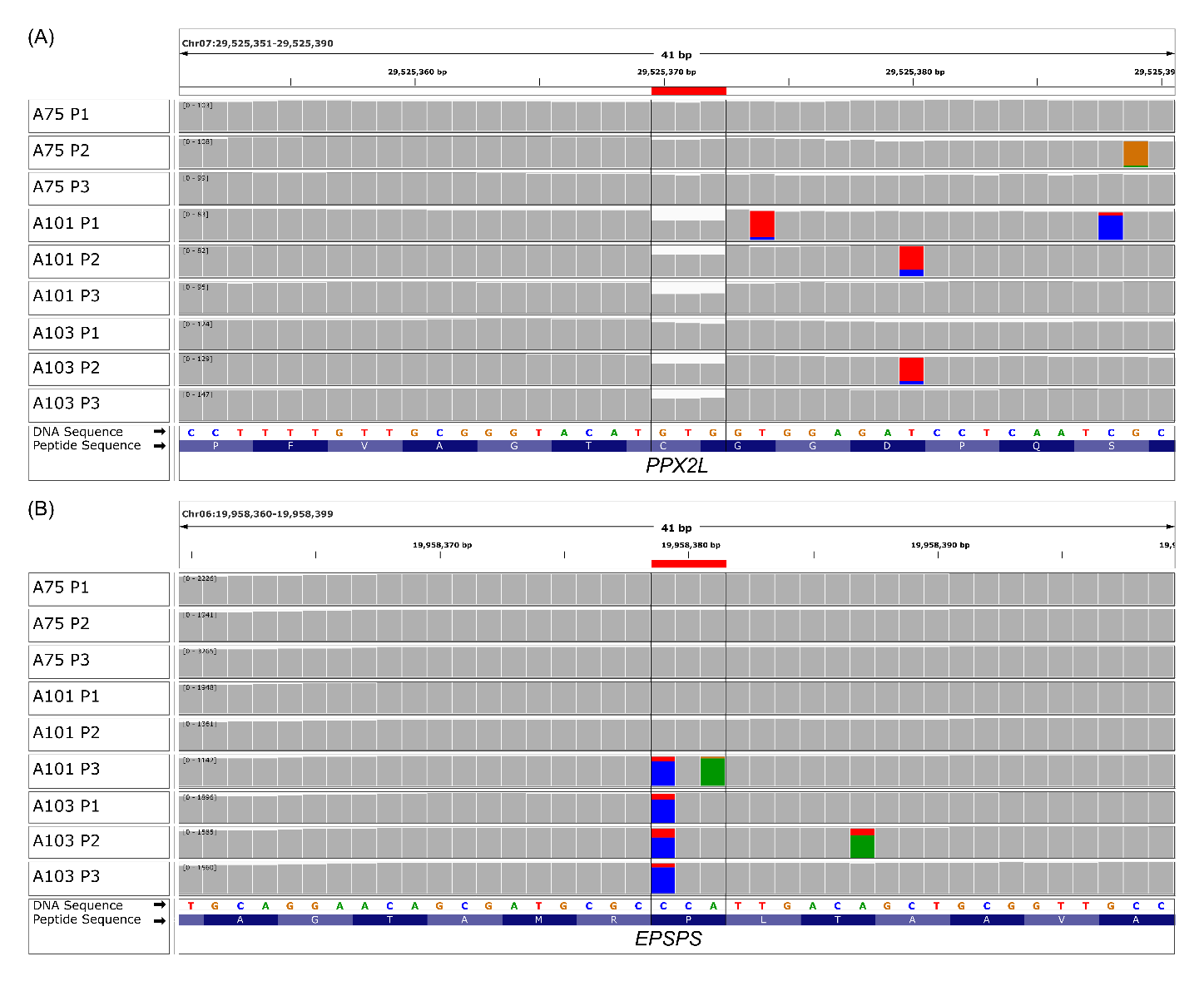


**Figure S6.** Transcriptome coverage plots across loci surrounding (A) *PPX2* ΔG210 and (B) *EPSPS* P106S residues involved in target-site resistance to PPO- and EPSPS-inhibitors, respectively. Bases at the site of ΔG210 deletion and P106S substitution are indicated by a red bar at the top and variants are colored by base according to their frequency in each pool.
